# Digitalis—Its Use in Suppression of Urine

**Published:** 1868-12-15

**Authors:** James T. Newman


### DIGITALIS, ITS USE IN SUPPRESSION OF URINE.

BY JAMES T. NEWMAN, M.D.

Was called, November 5th, 1868, to see a patient, who stated to me that he had been unable to pass his urine for two days. Vomiting and nausea had set in, with great pain in the lumbar regions. Also, severe headache, with a quick bounding pulse. Here evidently was a case of retention or suppression. I passed the catheter, in order to determine what was the matter; its introduction was not followed by the least particle of urine, then I knew that I had a case of suppression. Ordered him to have

℞ *Hydrargyri Chloridi*, gr. xij.
*Pill Gambogiæ*, Comp.
*Ext. Colocynth*, aa gr. xv.
*Syr. Zingiberis*, q. s.
*Misce fiant pilula*, No. x.

*Sig.* one every three hours, at the expiration of three hours to have a bottle *Citrate Magnesia.*

Visited him in the evening, found his bowels had been moved, but no urine. He was in great pain, and sinking rapidly. I ordered him to have a Turpentine bath; that is, to saturate a piece of flannel with *Spirits of Turpentine*, and apply to both sides of the spine. Take an iron, moderately warm, and bath the parts until the flannel is dry.

Ordered him to have

℞ *Potassæ Bitartratis*,

*Acidi Boracici*, aa ʒ ij.

*Aqua pura*, ℥ xij.

*Misce. Sig.* wine-glassful every hour. Left him for the night.

November 6th, found him no better than when first seen. No urine yet. My attention had been called to an article on the same subject, in Braithwaite, page 172, No. LVII., which spoke very highly of *Digitalis.* To tell the truth, when I commenced the practice of medicine, through a blunder of mine, I became prejudiced against *Digitalis.* I had determined in my mind never to use it. Here was the life of man in the way of my early ignorant convictions. I decided to give it a trial, ordered *Tr. Digitalis* ℥ ij., and a poultice of warm linseed meal, to be applied to the lower part of the abdomen. This was done at 8 o'clock in the morning; called to see him at three the same day, and found the urine flowing freely. Result, he made a rapid recovery.

*Case 2nd.* — November 20th, 1868, was called to see a gentleman similarly affected; no time was lost, gave him a brisk cathartic, and applied the *Digitalis* in the same manner as in case first; in six hours he made water with perfect ease. In presenting these facts to your many readers, it will be seen that I claim nothing new, but I do think that the drug deserves a trial.

